# Pharmacokinetic Evaluation of Clozapine in Concomitant Use of *Radix Rehmanniae*, *Fructus Schisandrae*, *Radix Bupleuri*, or *Fructus Gardeniae* in Rats

**DOI:** 10.3390/molecules21060696

**Published:** 2016-05-27

**Authors:** Dan-Dan Tian, Wei Wang, Hua-Ning Wang, Stephen Cho Wing Sze, Zhang-Jin Zhang

**Affiliations:** 1 School of Chinese Medicine, LKS Faculty of Medicine, the University of Hong Kong, Hong Kong, China; tiandd86@hotmail.com (D.-D.T.); wwei0825@gmail.com (W.W.); stephens@hku.hk (S.C.W.S.); 2 Department of Psychiatry, the fourth Military Medical University, Xi’an 710032, Shaanxi, China; xskzhu@fmmu.edu.cn

**Keywords:** herbal medicine, clozapine, pharmacokinetics, herb-drug interaction, metabolism

## Abstract

*Radix Rehmanniae*, *Fructus Schisandrae*, *Radix Bupleuri*, and *Fructus Gardeniae* are often used alongside with clozapine (CLZ) for schizophrenia patients in order to reduce side effects and enhance therapeutic efficacy. However, worse outcomes were observed raising concern about a critical issue, herb-drug interactions, which were rarely reported when antipsychotics were included. This study aims to determine whether the concomitant use of these herbal medicines affects the pharmacokinetic characteristics of CLZ in rat models. Rats were given a single or multiple intraperitoneal injections of 10 mg/kg CLZ, either alone or with individual herbal water extracts administered orally. CLZ and its two inactive metabolites, norclozapine and clozapine *N*-oxide, were determined by high-performance liquid chromatography/tandem mass spectrometry. In the acute treatment, the formation of both metabolites was reduced, while no significant change was observed in the CLZ pharmacokinetics for any of the herbal extracts. In the chronic treatment, none of the four herbal extracts significantly influenced the pharmacokinetic parameters of CLZ and its metabolites. Renal and liver functions stayed normal after the 11-day combined use of herbal medicines. Overall, the four herbs had limited interaction effect on CLZ pharmacokinetics in the acute and chronic treatment. Herb-drug interaction includes both pharmacokinetic and pharmacodynamic mechanisms. This result gives us a hint that pharmacodynamic herb-drug interaction, instead of pharmacokinetic types, may exist and need further confirmation.

## 1. Introduction

Clozapine (CLZ), a tricyclic dibenzodiazepine antipsychotic drug, is commonly prescribed for the long-term maintenance treatment of schizophrenia. CLZ is therapeutically effective for treating patients with schizophrenia that is refractory to other neuroleptics, without producing significant extrapyramidal symptoms. However, the associated adverse effects, such as hypersalivation, idiosyncratic hepatotoxicity, agranulocytosis, and cardiactoxicity, restrict its clinical use [1,2]. Herbal medicines are increasingly used in the treatment of schizophrenia to improve the therapeutic efficacy and reduce adverse side effects. Our previous epidemiological survey showed that approximately 37% of schizophrenia patients received antipsychotics in combination with herbal medicines, including *Radix Rehmanniae*, *Fructus Gardeniae*, *Radix Bupleuri*, and *Fructus Schisandrae*, *etc.* [3]. *Radix Rehmanniae* (RR, Di-Huang), derived from the roots of *Rehmannia glutinosa (Gaertn.) DC.*, is a traditional Chinese medicine frequently used for the treatment of anemia, diabetes, dizziness, tinnitus, nocturnal emission, and palpitation. According to traditional Chinese medicine theory, this herb can “nourish yin and replenish blood, reinforcing essence and marrow” [4]. In other words, it has a hemostatic effect, removing heat and enriching the blood [5]. *Fructus Schisandrae* (FS, Wu-Wei-Zi), the dried fruit from *Schisandra chinensis (Turcz.) Baill.*, is known to possess hepatoprotective and general detoxifying functions which is commonly used for the treatment of chronic cough and dyspnea, enuresis, frequent urination, and night sweating [6]. *Radix Bupleuri* (RB, Chai-Hu), the root of *Bupleurum chinense DC.*, is traditionally used for metabolic regulation and beneficial in treating fever, influenza, inflammation, malaria, and menstrual disorders [4,7]. *Fructus Gardeniae* (FG, Zhi-Zi), the dried fruit of *Gardenia jasminoides J. Ellis*, can suppress the evil fire, relieve internal heat, and cool the blood in the body according to Chinese medical theory. It is used to treat inflammation, jaundice, hepatitis, diabetes, atherosclerosis, cardiovascular diseases, and depression [4,8]. Because of these effects, the four herbs are commonly used in combination with CLZ by patients with schizophrenia. However, our previous study indicated that the concomitant use is, contrary to expectation, strongly associated with worse clinical outcomes [3]. The underlying mechanism is still unclear. Unfortunately, few reports focus on the interaction of herbal medicines and antipsychotics. Therefore, in this research, we evaluated the pharmacokinetic influence of herbs on clozapine metabolism.

Previous studies have found that CLZ is metabolized to norclozapine (norCLZ) and clozapine *N*-oxide (CLZ *N*-oxide) via cytochrome P450 (CYP) enzymes, mainly CYP1A2, CYP3A4, CYP2D6, CYP2C9, and CYP2C19 [9,10]. The metabolism and pharmacokinetics of CLZ may change if the activities of CYP enzymes are altered by disease or CYP modulators [11,12]. The concomitant use of rifampicin and St John’s wort, both of which induce CYP1A2 and CYP3A4, is known to decrease CLZ plasma levels in patients [13,14]. Oral contraceptives have been reported to cause elevated CLZ plasma levels due to CYP inhibition [15]. Therefore, the conjecture that metabolic inhibition or induction of CLZ takes place as a consequence of herbal addition seemed plausible. This would provide an explanation for the unfavorable therapeutic effects observed in clinic. 

The current study examined the influence of acute or chronic co-administration of the aforementioned four herbal preparations on CLZ pharmacokinetics in rats. Safety evaluation of 11-day combined use of herbal medicines on liver and kidney function were conducted by determination of the biochemical parameters, including aspartate aminotransferase (AST), alanine aminotransferase (ALT), creatinine (CR), and blood urea nitrogen (BUN) levels.

## 2. Results

### 2.1. Effects of Herbal Medicine on CLZ Pharmacokinetics in Rats

All calibration curves in biological samples exhibited good linearity. The accuracy calculated of bias% for CLZ, norCLZ and CLZ *N*-oxide were 16.8%, 14.7% and 15.1%, respectively. The precision calculated of RSD% were 9.1%, 9.1%, and 9.4% for CLZ, norCLZ, and CLZ *N*-oxide, respectively. The method sensitivity in plasma was determined by the LLOQ, which were 9.8, 13.6, and 17.1 nM for CLZ, norCLZ, and CLZ *N*-oxide, respectively. 

**Figure 1:**
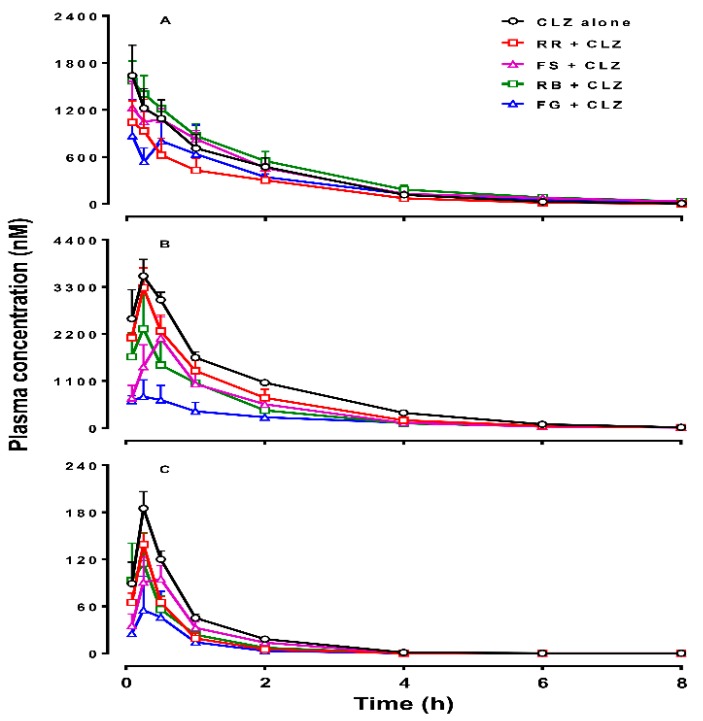
Plasma concentrations of clozapine (CLZ, **A**), norclozapine (norCLZ, B), and clozapine *N-*oxide (CLZ *N*-oxide, **C**) over time in rats treated with an intraperitoneal dose of 10 mg/kg of CLZ alone (*n* = 5), or pretreated orally with *Radix Rehmanniae* (RR; *n* = 4), *Fructus Schisandrae* (FS; *n* = 6), *Radix Bupleuri* (RB; *n* = 6), and *Fructus Gardeniae* (FG; *n* = 4), respectively. The data are presented as mean ± SEM.

**Figure 2:**
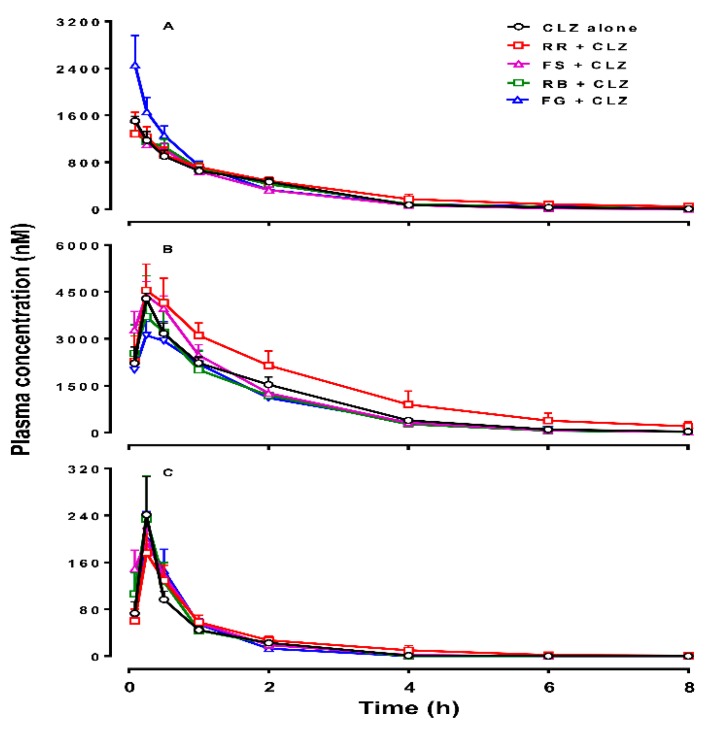
Plasma concentrations of clozapine (CLZ, **A**), norclozapine (norCLZ, **B**), and clozapine *N*-oxide (CLZ *N*-oxide, **C**) over time in rats treated with an intraperitoneal injection of 10 mg/kg of CLZ for 11 days alone (*n* = 5), or pretreated orally with *Radix Rehmanniae* (RR; *n* = 6), *Fructus Schisandrae* (FS; *n* = 4), *Radix Bupleuri* (RB; *n* = 4), and *Fructus Gardeniae* (FG; *n* = 4), respectively, every day. The data are presented as mean ± SEM.

Figure 1 and Figure 2 show the mean plasma concentrations of CLZ, norCLZ and CLZ *N*-oxide over time, following acute or chronic administration of CLZ in rats. The plasma pharmacokinetic parameters of the three compounds are summarized in Table 1 and Table 2.

In the acute CLZ treatment, RR cotreatment reduced the AUC_0-∞_ of CLZ *N*-oxide by approximately 50% (F = 3.596, *p* = 0.023), but did not significantly affect the AUC_0-∞_ of CLZ, norCLZ, or the *C*_max_ of CLZ (Table 1 and Figure 1). FS cotreatment reduced the AUC_0-∞_ and *C*_max_ values of norCLZ by 50% compared with CLZ alone (F = 3.902, *p* = 0.013), but did not significantly alter the systemic exposure of CLZ and CLZ *N*-oxide. The addition of RB extract reduced the AUC_0-∞_ of norCLZ by 51% (F = 3.902, *p* = 0.014) and the AUC_0-∞_ of CLZ *N*-oxide by 49% (F = 3.596, *p* = 0.012). A single-dose administration of FG extract decreased the AUC_0-∞_ of norCLZ by 75% (F = 3.902, *p* = 0.002), CLZ *N*-oxide by 73% (F = 3.596, *p* = 0.007), and CLZ by 21%. The *C*_max_ value of CLZ *N*-oxide was reduced by 31% (F = 2.638, *p* = 0.008) after adding FG.

The values of concentration ratios of norCLZ to CLZ (norCLZ/CLZ) at 0.25 h post-dose in the presence of FS and RB and the values of CLZ *N*-oxide/CLZ at 1 h post-dose in the presence of RB and 2 h post-dose in the presence of RB and FG were significantly different from that in the control group. The other metabolism ratios (plasma concentration of either metabolite to CLZ) between the herbal addition groups and CLZ alone group were comparable (Table S1 in Supplementary Materials). In the chronic treatment, the combination of individual herbal extracts did not significantly change the kinetic parameters of CLZ, norCLZ, or CLZ *N*-oxide (Table 2). Despite that, co-administration of RR with CLZ for 11 days increased the mean AUC_0-∞_ value of CLZ, norCLZ, and CLZ *N*-oxide by 33%, 60%, and 37%, respectively. Figure 2 shows that the plasma concentrations of CLZ in the FG group were higher than those in the control group at 5, 15, and 30 min. However, there was no difference in metabolism ratios of either metabolite to CLZ between the groups treated with and without herbal medicines (Table S2). Comparison of the pharmacokinetic parameters of CLZ, norCLZ, and CLZ *N*-oxide between the groups of acute and chronic administration of CLZ alone showed no self-induction/inhibition and there was no accumulation of the three compounds in the rats, consistent with a previous report [16].

### 2.2. Biodistribution of CLZ in Various Organs

The LLOQ in all the tissues was 3.28 nM for CLZ, 5.45 nM for norCLZ and 29.8 nM for CLZ *N*-oxide, respectively. Rat organs were collected 24 h after the last dose in the long-term treatment group. CLZ and its metabolites were almost undetectable in the heart, liver, spleen, lung, kidney, and whole brain, which were in parallel with the plasma levels.

### 2.3. Safety Evaluation of Chronic Administration of Herbs on Rat Liver and Renal Functions

**Figure 3:**
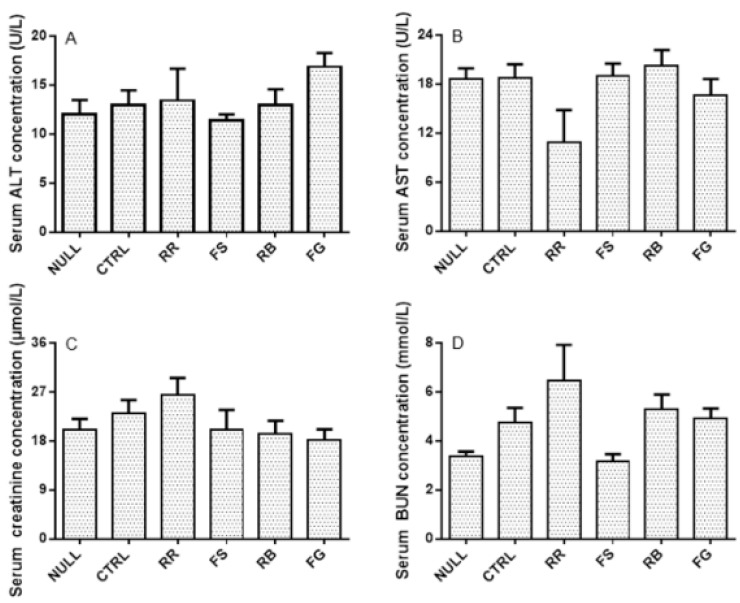
Serum concentrations of alanine aminotransferase (ALT; **A**), aspartate aminotransferase (AST; **B**), creatinine (CR; **C**), and blood urea nitrogen (BUN; **D**) after 11-day treatment of vehicle (CTRL) and clozapine (CLZ) with or without *Radix Rehmanniae* (RR), *Fructus Schisandrae* (FS), *Radix Bupleuri* (RB), and *Fructus Gardeniae* (FG), respectively. The data are presented as mean ± SEM.

Comparison of the serum AST, ALT, CR, and BUN levels between the herbal additive groups and the CLZ alone group indicated that rat liver and renal functions were not affected by the four herbs in the chronic study (Figure 3).

## 3. Discussion

Previous studies have demonstrated that schizophrenia patients have a higher prevalence of cardiovascular disease, diabetes mellitus, and metabolic syndrome than the general population, while antipsychotic drugs can aggravate glucose and lipid metabolism disorders [17,18]. Combined administration of herbs and drug has enjoyed increasing popularity in recent decades. RR, FS, RB, and FG are widely used in psychiatric patients for relieving adverse effects and improving efficacy. It has been reported that the administration of FS and RR in combination with other herbs has the potential to treat various cardiovascular diseases [19] and improve serum lipid and glucose levels [20]. An animal study with vinegar-baked RB indicated its effect on the regulation of lipid disorders by increasing fatty acid oxidation [21]. Geniposide from FG was confirmed to alleviate insulin resistance and abnormal lipid metabolism [22]. Crocetin and crocin from a water extract of FG showed anti-hypertensive effects [8]. However, the concomitant use of certain herbs and antipsychotics did not reach the intended outcome. On the contrary, worse clinical outcomes were observed [3]. Therefore, disclosure of the underlying mechanisms is urgently required. The altered efficacy of drugs can be due to herb-drug interaction which includes both pharmacokinetic and pharmacodynamic interactions. This study investigated the effect of co-administration of individual water extracts of four herbs on the pharmacokinetics of an effective atypical antipsychotic agent, CLZ, in rats.

It has been reported that the four herbs investigated in this study, RR, FS, RB, and FG, have the potential to inhibit CYP activitities. RR was reported to inhibit the activity of CYP1A2, CYP2C9, CYP2D6, CYP2E1, and CYP3A4 using probe substrates *in vitro* [23]. Vinegar-baked RB was shown to significantly inhibit the activity of CYP2C9 but not that of CYP1A2 or CYP3A4 [24]. Genipin, an aglycone of geniposide from FG, is a main component exhibiting pharmacological activity that can significantly induce CYP2D6 enzyme, and inhibit CYP2C19 and CYP3A4 activity [25]. A single dose of FS in rats was reported to have an inhibitory effect on CYP1A2 and CYP3A4 and an inducible effect on CYP2E1, using theophylline, midazolam, and dapsone as probe substrates [26,27]. As CLZ is mainly metabolized by CYPs, the four herbs may affect CLZ pharmacokinetics via CYP enzyme modulation.

In the acute study, the formation of norCLZ and CLZ *N*-oxide dropped to various extents when CLZ was used in combination with individual herbs. The greatest reduction in the AUC_0-∞_ of norCLZ was observed in the FG group (75%), followed by the RB (51%) and FS groups (50%). The decreased systemic exposure of CLZ metabolites suggested CYP enzyme inhibition by FS, RB, and FG in CLZ metabolism, which is consistent with the reported inhibitory effect of these four herbs. However, the decreased extent of CLZ metabolism is due to the cooperative contribution of each isozyme, the intrinsic inhibitory potential of and *in vivo* concentrations of active components from herbs. The systemic exposure of CLZ showed no significant difference among different groups. ClZ was metabolized via oxidation, sulfation, and glucuronidation to hydroxylated metabolites, CLZ-glucuronides, norCLZ-glucuronides as well as the two major metabolites norCLZ and CLZ *N*-oxide [28]. Compensatory metabolism of other metabolic pathways with the addition of herb extract may lead to the same CLZ level in the acute study. NorCLZ is a metabolite with limited pharmacological activity and CLZ *N*-oxide had little activity. Clinical outcomes are reported to be related to the concentration ratios of norCLZ to CLZ [29]. However, the norCLZ/CLZ ratio remained unchanged except at 0.25 h after dosing with CLZ and FS or RB in the acute study. It could be concluded that the four herbs tested played very limited role in causing pharmacokinetic herb-CLZ interaction.

Studies of herb-drug interactions are commonly performed with a single bolus dose, whereas the clinical use of herbs and drugs often lasts for longer periods. Therefore, in the chronic study, the herbal medicines were repeatedly administered at their human equivalent doses over an 11-day period to mimic clinical usage. No statistically significant differences in the pharmacokinetics of CLZ were observed in the presence and absence of the herbal medicines, although there was a trend of increased systemic exposure of CLZ with RR and FG addition, of norCLZ with RR addition, and of CLZ *N*-oxide with each herbal addition. It has been reported that multiple doses of FS can induce CYP3A4 activity but decrease CYP1A2 and CYP2E1 activity in rats using probe substrates [26,27]. One-week administration of ethanol/water extraction of RB robustly induced CYP2E1, CYP2D6, and CYP3A4 when chlorzoxazone, dextromethorphan, and midazolam were chosen as substrates, respectively. However, RB had no significant effect on CYP1A2, CYP2C9, and CYP2C19 when caffeine, tolbutamide, and omeprazole were selected as probe substrates, respectively [30]. Collectively, multiple components contained in each herb may have different effects on CYP isozymes. Moreover, the low systemic exposure of bioactive components in herbs may lead to a lack of modulation of CYP enzymes *in vivo*. In summary, it seems impossible that the four herbs would cause an interaction with CLZ pharmacokinetics.

## 4. Experimental Section

### 4.1. Drugs and Reagents

CLZ and its two metabolites, norCLZ and CLZ *N*-oxide, were obtained from Selleck Chemicals (Houston, TX, USA), Tocris (Bristol, UK), and Enzo (New York, NY, USA), respectively. Olanzapine was purchased from Meryer Chemical Technology Co., Ltd. (Shanghai, China). Bioactive compounds contained in the four herbs including saikosaponin A, saikosaponin D, geniposide, gardein A, catalpol, acteoside, schisandrin, and schisandrol B were purchased from Shanghai Yuanye Bio-Technology Co., Ltd. (Shanghai, China). ALT and AST Elisa kits were bought from Yuan Ye Bio-Technology Co., Ltd. (Shanghai, China). CR and BUN assay kits were purchased from Jian Cheng Chemical Industrial Co., Ltd. (Nanjing, China). Heparin sodium salt was obtained from Sigma Aldrich. High-performance liquid chromatography (HPLC)-grade actonitrile was supplied by Duksan Pure Chemical Co., Ltd. (Ansan, South Korea) and other analytical reagents by Sigma Aldrich (St. Louis, MO, USA).

### 4.2. Herbal Extraction Preparation

Raw materials of *Radix Rehmanniae* (RR, Di-Huang), *Fructus Schisandrae* (FS, Bei Wu-Wei-Zi), *Radix Bupleuri* (RB, Chai-Hu), and *Fructus Gardeniae* (FG, Zhi-Zi) were supplied and deposited by the pharmacy of the School of Chinese Medicine at the University of Hong Kong (Hong Kong, China). The specimens were identified before use by Dr. Yan-Bo Zhang at the University of Hong Kong. The extraction was conducted as recommended in the Chinese Pharmacopoeia to preserve the bioactive constituents. Briefly, the raw material of each herb (1.8 kg) was sliced, broiled, immersed, and boiled in a 10-fold volume of distilled water for 2 h. The residue was then extracted by repeating the same process, as previously reported [31]. The extracted solution was pooled and concentrated using a vacuum rotary evaporator. The four herbs used in the present work are well known plants with fully known active compounds [4]: catalpol and acteoside from RR, schisandrin and chisandrol B from FS, saikosaponin A and saikosaponin D from RB, geniposide and gardenin A from FG. Therefore, the eight compounds were selected as marker components and measured using HPLC. The contents of catalpol, acteoside, schisandrin, schisandrol B, saikosaponin A, geniposide, and gardenin A were 5.60 ± 0.28, 1.59 ± 0.24, 0.65 ± 0.05, 1.84 ± 0.37, 0.39 ± 0.05, 48.3 ± 12.0, 0.32 ± 0.02 (mean ± SD, *n* = 3) mg/g raw material [32]. The content of saikosaponin D in the water extraction was too low to quantitate. A stock solution of 1 g raw material/mL was prepared for each herb. 

### 4.3. Rat Pharmacokinetic Studies

All rat studies were approved by the Committee on the Use of Live Animals in Teaching and Research of the LKS Faculty of Medicine at the University of Hong Kong. Male Sprague-Dawley rats with a body weight of 250 ± 20 g were housed in an animal room at 23 ± 1 °C with a 12-h light/dark cycle (lights on 06:00–18:00 h). Water and food were available *ad libitum*. In the acute treatment, the rats were randomly assigned to five groups (at least four rats per group) and administered with CLZ alone or in combination with RR (15 g/kg), FS (5 g/kg), RB (5 g/kg), or FG (8 g/kg). Following an overnight fast, the rats were injected intraperitoneally with CLZ at 10 mg/kg alone or 15 min after oral administration of each herbal preparation. CLZ was intraperitoneally injected to avoid the influence on its intestinal absorption by herbal addition and to investigate the underlying mechanism with respect of metabolism. The 15-min interval was set to produce high concentrations of herbal constituents when CLZ was injected to rats. Individual herbal preparations were administered at 5 to 8 times the clinical dose. Serial blood samples (0.3 mL each) were collected from the jugular vein at 0, 0.083, 0.25, 0.5, 1, 2, 4, 6, 8, and 24 h post-treatment. In the chronic study, the rats were randomly assigned to five groups (at least four rats per group) and administered with CLZ alone, or in combination with RR (3 g/kg), FS (1 g/kg), RB (1 g/kg), or FG (1 g/kg). The dosages of these herbal preparations were equivalent to the dosages used in clinical practice. Rats were injected intraperitoneally with CLZ at 10 mg/kg alone or in combination with orally administered individual herbal preparations once daily for 11 consecutive days. Serial blood samples (0.3 mL each) were collected from the jugular vein at 0, 0.083, 0.25, 0.5, 1, 2, 4, 6, 8, and 24 h after the last dose. After centrifugation, the obtained plasma samples were stored at −80 °C until further analysis. During sample collection period, 0.3 mL sterile isotonic saline containing 20 IU/L heparin was injected through jugular vein after sampling at each time point to compensate for the blood loss.

After blood samples were taken from the rats receiving long-term treatment, the rats were sacrificed and trunk blood, heart, liver, spleen, lung, kidney, and brain were obtained. Blood was also collected from the rats receiving no drug treatment and served as control. Tissues were homogenized with addition of 4 volumes of iced water. Serum samples were stored at −80 °C until determination of AST, ALT, CR, and BUN levels and tissue homogenate were stored at −80 °C for analysis of CLZ and its metabolites.

### 4.4. Biochemical Assay of AST, ALT, CR, and BUN Levels in Chronic Treated Rats

All of the procedures for determining AST, ALT, CR, and BUN levels were exactly the same as the protocols provided by the manufacturers.

### 4.5. The Measurement of CLZ, norCLZ, and CLZ N-oxide

Plasma and tissue homogenates were deproteinized and the mixed solution was vortexed and centrifuged [33]. The supernatants were obtained and an aliquot of 10 μL of each sample was injected to the LC-MS/MS system. CLZ and its metabolites were measured using an API 3200 Qtrap mass spectrometer (Applied Biosystems, Foster City, CA, USA) connected with an Agilent 1200 HPLC system (Agilent Technologies, Palo Alto, CA, USA). An ACE 5 AQ column (5 μm, 4.6 × 250 mm) maintained at room temperature was applied for chromatographic separation. The mobile phase consisted of acetonitrile (A) and water containing 0.1% formic acid (B) and was delivered at 0.7 mL/min following a gradient program: 40% to 95% A (0–7.0 min), 95% (7.0–8.0 min), 95% to 40% A (8.0–8.1 min), and 40% A (8.1–11 min). The precursor-to-product ion pairs used for monitoring the multiple reactions of CLZ, CLZ *N*-oxide, and norCLZ were *m*/*z* 327.2→270.1, 343.2→192.1, and 313.2→192.1, respectively. Olanzapine was selected as the internal standard. Analyst software version 1.5.2 (Applied Biosystems, Foster City, CA, USA) was used for the HPLC-MS/MS system operation, data acquisition, and processing. Validation of the method was conducted. Only intra-day precision and accuracy were provided because biological samples were analyzed within one day every time.

### 4.6. Data Analysis

Pharmacokinetic parameters were determined using the Kinetica program ((InnaPhase Corp., Philadelphia, PA, USA). The peak concentration (*C*_max_), time to reach *C*_max_ (*t*_max_), area under the concentration-time curve between time 0 and infinity (AUC_0-∞_), elimination half-life (*t*_1/2_), total body clearance (CL/F), mean residence time (MRT), and volume of distribution (*V*_d_) were obtained. All data are expressed as the mean ± the standard error of the mean (SEM). Biochemical data were analyzed using one-way analysis of variance (ANOVA) followed by post hoc multiple comparisons. Fisher’s Least Significant Difference (LSD) test was performed for multiple comparisons if equal variance was assumed; otherwise, the Dunnet T3 method was applied. *p* < 0.05 was considered to be the minimum level of statistical significance.

## 5. Conclusions

The present study demonstrated that an acute dose of individual water preparation of RR, FS, RB, and FG inhibited CLZ metabolism in rats but both acute and chronic combination use of the herbal medicines did not significantly affect the pharmacokinetics of CLZ. Our study indicates that CLZ efficacy was little influenced by pharmacokinetic interaction between the abovementioned herbal medicines and CLZ in rats. These results provide valuable information for our understanding of why combined use of RR, FS, RB, or FG and CLZ gives worse results. Other mechanisms, like pharmacodynamic interaction between herbs and CLZ, should be evaluated in the future. Further studies with human subjects are also needed to confirm the results in rats.

## Figures and Tables

**Table 1:** Effects of acute cotreatment with *Radix Rehmanniae* (RR), *Fructus* *Schisandrae* (FS), *Radix Bupleuri* (RB), and *Fructus Gardeniae* (FG) on the pharmacokinetics of plasma clozapine, norclozapine (norCLZ), and clozapine *N*-oxide (CLZ *N*-oxide) in rats after an intraperitoneal dose of 10 mg/kg clozapine ^a,b^.

Variable ^b^	CLZ Alone (*n* = 5)	RR + CLZ (*n* = 4)	FS + CLZ (*n* = 6)	RB + CLZ (*n* = 6)	FG + CLZ (*n* = 4)
CLZ					
*C*_max_ (nM)	1721 ± 349	1118 ± 350	1434 ± 232	1807 ± 205	1344 ± 469
*T*_max_ (h)	0.20 ± 0.12	0.12 ± 0.042	0.40 ± 0.14	0.28 ± 0.08	0.36 ± 0.14
AUC_0-C6_ (nM∙h)	2370 ± 526	1418 ± 543	2640 ± 350	2992 ± 509	1871 ± 649
*t*_1/2_ (h)	1.16 ± 0.091	1.01 ± 0.7	2.50 ± 1.06	1.17 ± 0.15	2.09 ± 0.99
MRT (h)	1.57 ± 0.053	1.45 ± 0.085	2.46 ± 0.74	1.82 ± 0.30	2.68 ± 1.17
CL/F (L/h/kg)	16.7 ± 4.43	30.1 ± 8.06	12.9 ± 2.01	11.7 ± 1.77	55.2 ± 41.8
*V*_d_ (L)	27.1 ± 6.36	43.6 ± 10.9	39.0 ± 12.5	18.3 ± 1.72	94.0 ± 50.3
NorCLZ					
*C*_max_ (nM)	3644 ± 426	3283 ± 461	1996 ± 620	2364 ± 879	768 ± 379 (0.008) *
*T*_max_ (h)	0.27 ± 0.07	0.25 ± 0	0.67 ± 0.27	0.64 ± 0.30	1.15 ± 0.95
AUC_0-C5_ (nM∙h)	5819 ± 382	4238 ± 875	2862 ± 878 (0.013) *	2878 ± 908 (0.014) *	1463 ± 619 (0.002) *
*t*_1/2_ (h)	1.08 ± 0.09	1.05 ± 0.10	1.20 ± 0.19	1.08 ± 0.16	1.5 ± 0.38
MRT (h)	1.66 ± 0.10	1.34 ± 0.10	1.75 ± 0.39	1.97 ± 0.47	2.61 ± 0.86
CLZ *N*-oxide					
*C*_max_ (nM)	185 ± 21.3	139± 14.0	112 ± 28.2	134 ± 47.2	58.2 ± 42.8 (0.008) *
*T*_max_ (h)	0.25± 0	0.25 ± 0	0.46 ± 0.12	0.64 ± 0.30	0.21 ± 0.11
AUC_0-C1_ (nM∙h)	159 ± 16.2	82.5 ± 10.9 (0.023) *	130 ± 14.2	81.6 ± 23.4 (0.012) *	43.1 ± 33.1 (0.007) *
*t*_1/2_ (h)	0.69 ± 0.12	0.39 ± 0.09	1.04 ± 0.44	0.39 ± 0.04	0.59 ± 0.12
MRT (h)	0.95 ± 0.15	0.57 ± 0.13	1.43 ± 0.61	0.79 ± 0.15	0.70 ± 0.21

^a^. Data are expressed as mean ± SEM and analyzed using one-way ANOVA. * *P* value compared with the CLZ alone group; ^b^. *C*_max_, peak concentration; *T*_max_, time to reach *C*_max_; AUC_0-∞_, area under the concentration-time curve from time zero to infinity; *t*_1/2_, elimination half-life; CL/F, total body clearance; MRT, mean residence time; *V*_d_, volume of distribution.

**Table 2:** Effects of chronic co-treatment with *Radix Rehmanniae* (RR), *Fructus Schisandrae* (FS), *Radix Bupleuri* (RB), and *Fructus Gardeniae* (FG) on the pharmacokinetics of plasma clozapine, norclozapine (norCLZ), and clozapine *N*-oxide (CLZ *N*-oxide) in rats after intraperitoneal administration of 10 mg/kg clozapine for 11 days ^a,b^.

Variable ^b^	CLZ Alone (*n* = 5)	RR + CLZ (*n* = 6)	FS + CLZ (*n* = 4)	RB + CLZ (*n* = 4)	FG + CLZ (*n* = 4)
CLZ					
*C*_max_ (nM)	1430 ± 122	1499 ± 280	1537 ± 118	1330 ± 125	2493 ± 472
*T*_max_ (h)	0.15 ± 0.04	0.21 ± 0.07	0.08 ± 0	0.19 ± 0.10	0.12 ± 0.04
AUC_0-∞_ (nM∙h)	2039 ± 211	2710 ± 545	1886 ± 218	2190 ± 408	2429 ± 138
*t*_1/2_ (h)	1.36 ± 0.23	2.40 ± 0.82	1.26 ± 0.47	2.19 ± 1.10	1.25 ± 0.21
MRT (h)	1.63 ± 0.13	2.43 ± 0.59	1.31 ± 0.07	1.75 ± 0.46	1.39 ± 0.21
CL/F (L/h/kg)	15.5 ± 1.30	14.1 ± 3.10	17.0 ± 2.19	15.4 ± 2.57	12.8 ± 0.75
*V*_d_ (L)	26.5 ± 50.2	33.0 ± 9.48	30.9 ± 11.9	38.3 ± 11.8	21.5 ± 2.66
NorCLZ					
*C*_max_ (nM)	4050 ± 268	4677 ± 807	4383 ± 444	3754 ± 1266	3200 ± 528
*T*_max_ (h)	0.30 ± 0.05	0.29 ± 0.04	0.25 ± 0	0.31 ± 0.06	0.31 ± 0.06
AUC_0-∞_ (nM∙h)	7192 ± 762	11494 ± 2767	7347 ± 1237	6125 ± 1738	6078 ± 911
*t*_1/2_ (h)	2.08 ± 0.49	1.72 ± 0.23	1.93 ± 0.15	1.80 ± 0.16	1.39 ± 0.19
MRT (h)	1.82 ± 0.14	2.19 ± 0.43	1.48 ± 0.12	1.83 ± 0.45	1.73 ± 0.06
CLZ *N*-oxide					
*C*_max_ (nM)	214 ± 57.4	191 ± 32.7	196 ± 22.0	237 ± 69.5	200 ± 46.9
*T*_max_ (h)	0.30 ± 0.05	0.29 ± 0.04	0.25 ± 0	0.31 ± 0.06	0.25 ± 0
AUC_0-∞_ (nM∙h)	146 ± 20.1	199 ± 48.5	182 ± 7.41	172 ± 39.9	159 ± 34.0
*t*_1/2_ (h)	0.55 ± 0.05	0.87 ± 0.12	0.61 ± 0.03	0.63 ± 0.12	0.44 ± 0.02
MRT (h)	0.91 ± 0.11	1.26 ± 0.22	0.82 ± 0.04	1.00 ± 0.26	0.72 ± 0.02

^a^. Data are expressed as mean ± SEM and analyzed using one-way ANOVA. ^b^. *C*_max_, peak concentration; *T*_max_, time to reach *C*_max_; AUC_0-∞_, area under the concentration-time curve from time zero to infinity; *t*_1/2_, elimination half-life; CL/F, total body clearance; MRT, mean residence time; *V*_d_, volume of distribution.

## Supplementary Materials

Supplementary materials can be accessed at: http://www.mdpi.com/1420-3049/21/6/696/s1.

## Abbreviations

CLZ, clozapine; norCLZ, norclozapine; CLZ *N*-oxide, clozapine *N*-oxide; CYPs, cytochrome P450s; RR, *Radix Rehmanniae* (Di-Huang); FS, *Fructus Schisandrae* (Wu-Wei-Zi); RB, *Radix Bupleuri* (Chai-Hu); FG, *Fructus Gardeniae* (Zhi-Zi); HPLC/MS/MS, high-performance liquid chromatography/tandem mass spectrometry; *C*_max_, peak concentration; *T*_max_, time to reach *C*_max_; AUC_0-∞_, area under the concentration-time curve from time zero to infinity; *t*_1/2_, elimination half-life; CL/F, total body clearance; MRT, mean residence time; *V*_d_, volume of distribution.
