# Supplementary material for: Pharmacokinetic Evaluation of Clozapine in Concomitant Use of Radix Rehmanniae, Fructus Schisandrae, Radix Bupleuri, or Fructus Gardeniae in Rats
Source: Molecules. 2016 May 27;21(6):696. doi: 10.3390/molecules21060696 (PMC6272930; doi:10.3390/molecules21060696)
Supplement: Supplementary file 1 [file molecules-21-00696-s001.pdf]

Supplementary Materials: Pharmacokinetic Evaluation of Clozapine in Concomitant Use of *Radix Rehmanniae*, *Fructus Schisandrae*, *Radix Bupleuri*, or *Fructus Gardeniae* in Rats

Dan-Dan Tian, Wei Wang, Hua-Ning Wang, Stephen Cho Wing Sze and Zhang-Jin Zhang

Table S1. Metabolism ratios at different sample collection times after an intraperitoneal dose of 10 mg/kg clozapine to rats with or without *Radix Rehmanniae* (RR), *Fructus Schisandrae* (FS), *Radix Bupleuri* (RB), or *Fructus Gardeniae* (FG) [a].

Tiime (h)	CLZ alone (*n* = 5)	RR + CLZ (*n* = 4)	FS + CLZ (*n* = 6)	RB + CLZ (*n* = 6)	FG + CLZ (*n* = 4)
NorCLZ/CLZ					
0.083	1.90 ± 0.59	2.51 ± 0.67	0.56 ± 0.30	0.93 ± 0.33	3.65 ± 3.15
0.25	3.68 ± 1.01	4.63 ± 0.86	1.36 ± 0.46 (0.044) *	1.41 ± 0.47 (0.049) *	2.23 ± 1.34
0.5	3.55 ± 0.96	4.22 ± 0.63	1.85 ± 0.24	1.29 ± 0.43	1.55 ± 0.84
1	3.35 ± 1.05	3.58 ± 0.46	1.54 ± 0.66	1.45 ± 0.5	2.14 ± 1.34
2	3.17 ± 0.97	2.84 ± 0.49	1.48 ± 0.56	1.08 ± 0.32	0.91 ± 0.56
4	4.79 ± 1.95	2.72 ± 0.42	1.36 ± 0.44	0.97 ± 0.28	2.07 ± 1.12
6	1.80 ± 0.23	2.29 ± 0.08	1.05 ± 0.40	0.89 ± 0.27	1.25 ± 0.51
CLZ *N*-oxide/CLZ					
0.083	0.07 ± 0.02	0.09 ± 0.03	0.03 ± 0.02	0.05 ± 0.03	0.14 ± 0.12
0.25	0.19 ± 0.05	0.21 ± 0.05	0.09 ± 0.03	0.08 ± 0.03	0.12 ± 0.07
0.5	0.14 ± 0.03	0.13 ± 0.02	0.09 ± 0.01	0.05 ± 0.02	0.10 ± 0.06
1	0.08 ± 0.02	0.04 ± 0	0.05 ± 0.02	0.03 ± 0.01 (0.046) *	0.03 ± 0.03
2	0.04 ± 0.01	0.02 ± 0	0.04 ± 0.01	0.01 ± 0 (0.025) *	0.01 ± 0.01 (0.036) *
4	0.01 ± 0.01	0	0	0	0
6	0	0	0	0	n/a [b]

[a]. Data are expressed as mean ± SEM and analyzed using one-way ANOVA. * p value compared with the CLZ alone group. [b]. Not applicable.

Table S2. Metabolism ratios at different sample collection times after intraperitoneal administration of 10 mg/kg clozapine to rats for 11 days with or without *Radix Rehmanniae* (RR), *Fructus Schisandrae* (FS), *Radix Bupleuri* (RB), or *Fructus Gardeniae* (FG) [a].

Time (h)	CLZ Alone (*n* = 5)	RR + CLZ (*n* = 6)	FS + CLZ (*n* = 4)	RB + CLZ (*n* = 4)	FG + CLZ (*n* = 4)
NorCLZ/CLZ					
0.083	1.54 ± 0.29	3.14 ± 1.59	2.11 ± 0.23	2.03 ± 0.74	0.83 ± 0.13
0.25	3.46 ± 0.54	4 ± 0.73	3.99 ± 0.21	3.27 ± 1.14	1.96 ± 0.33
0.5	3.62 ± 0.43	4.81 ± 0.94	3.99 ± 0.4	3.41 ± 1.23	2.37 ± 0.42
1	3.53 ± 0.42	4.63 ± 0.94	3.92 ± 0.35	3.34 ± 1.2	2.95 ± 0.29
2	3.32 ± 0.37	4.72 ± 1.14	3.88 ± 0.47	3.02 ± 0.92	3.33 ± 0.24
4	5.58 ± 1.15	6.35 ± 1.96	4.42 ± 0.86	4.34 ± 1.43	4.13 ± 0.56
6	4.66 ± 0.98	6.75 ± 2.84	7.78 ± 1.88	3.52 ± 0.93	3.86 ± 1.16
CLZ N-oxide/CLZ					
0.083	0.06 ± 0.01	0.08 ± 0.04	0.10 ± 0.02	0.09 ± 0.03	0.03 ± 0.01
0.25	0.23 ± 0.05	0.15 ± 0.02	0.18 ± 0.02	0.21 ± 0.07	0.12 ± 0.03
0.5	0.12 ± 0.03	0.15 ± 0.03	0.14 ± 0.02	0.13 ± 0.04	0.12 ± 0.03
1	0.07 ± 0.01	0.08 ± 0.01	0.09 ± 0.02	0.07 ± 0.02	0.08 ± 0.02
2	0.05 ± 0.01	0.06 ± 0.01	0.06 ± 0	0.05 ± 0.01	0.04 ± 0.01
4	0.02 ± 0.01	0.03 ± 0.01	0.03 ± 0.01	0	0
6	0	0.01 ± 0.01	0	0	0

[a]. Data are expressed as mean ± SEM and analyzed using one-way ANOVA.
